# Supplementary material for: Whole Genome Sequencing of DENV-2 isolated from Aedes aegypti mosquitoes in Esmeraldas, Ecuador. Genomic epidemiology of genotype III Southern Asian-American in the country
Source: bioRxiv. 2024 Feb 8:2024.02.06.579255. Preprint. [Version 1] doi: 10.1101/2024.02.06.579255 (PMC10871324; doi:10.1101/2024.02.06.579255)
Supplement: Supplement 1 [file NIHPP2024.02.06.579255v1-supplement-1.pdf]

bioRxiv preprint doi: https://doi.org/10.1101/2024.02.06.579255; this version posted February 8, 2024. The copyright holder for this preprint (which was not certified by peer review) is the author/funder, who has granted bioRxiv a license to display the preprint in perpetuity. It is made available under aCC-BY 4.0 International license.

Supporting information

S1 Table. Specific primers designed for sequencing DENV-2. List of primers and sequences used for Illumina MiSeq sequencing.

S2 Table. Sequenced positive pool data. Information of female *Aedes aegypti* mosquitoes corresponding to the sequenced pool.

S1 Fig. Phylogenetic tree generated in MSA to compare our sample with other countries. The Ecuadorian sample reported in this study is genetically closer to samples collected from Colombia and Venezuela.
